# Coenzyme Q Depletion Reshapes MCF-7 Cells Metabolism

**DOI:** 10.3390/ijms22010198

**Published:** 2020-12-28

**Authors:** Wenping Wang, Irene Liparulo, Nicola Rizzardi, Paola Bolignano, Natalia Calonghi, Christian Bergamini, Romana Fato

**Affiliations:** Department of Pharmacy and Biotechnology, FABIT, University of Bologna, 6, 40126 Bologna, Italy; ww363@cinj.rutgers.edu (W.W.); irene.liparulo2@unibo.it (I.L.); nicola.rizzardi2@unibo.it (N.R.); paola.bolignano@studio.unibo.it (P.B.); natalia.calonghi@unibo.it (N.C.); romana.fato@unibo.it (R.F.)

**Keywords:** mitochondrial dysfunction, glycolysis, bioenergetics, glutamine metabolism, metabolic reprogramming, cancer metabolism targeting, coenzyme Q, spheroids

## Abstract

Mitochondrial dysfunction plays a significant role in the metabolic flexibility of cancer cells. This study aimed to investigate the metabolic alterations due to Coenzyme Q depletion in MCF-7 cells. Method: The Coenzyme Q depletion was induced by competitively inhibiting with 4-nitrobenzoate the coq2 enzyme, which catalyzes one of the final reactions in the biosynthetic pathway of CoQ. The bioenergetic and metabolic characteristics of control and coenzyme Q depleted cells were investigated using polarographic and spectroscopic assays. The effect of CoQ depletion on cell growth was analyzed in different metabolic conditions. Results: we showed that cancer cells could cope from energetic and oxidative stress due to mitochondrial dysfunction by reshaping their metabolism. In CoQ depleted cells, the glycolysis was upregulated together with increased glucose consumption, overexpression of GLUT1 and GLUT3, as well as activation of pyruvate kinase (PK). Moreover, the lactate secretion rate was reduced, suggesting that the pyruvate flux was redirected, toward anabolic pathways. Finally, we found a different expression pattern in enzymes involved in glutamine metabolism, and TCA cycle in CoQ depleted cells in comparison to controls. Conclusion: This work elucidated the metabolic alterations in CoQ-depleted cells and provided an insightful understanding of cancer metabolism targeting.

## 1. Introduction

Tumour cells opportunistically utilize various metabolic profiles depending on their genotypes [1,2,3], microenvironment [4,5,6], oxygen and substrate availability [7] and cellular signalling [8,9], to cope with bioenergetic, biosynthetic and redox demand [10].

Since some tumour cells presented the so-called Warburg effect, characterized by a high rate of glucose uptake and lactate production, even in the presence of oxygen, it was assumed that cancer cells had defective mitochondrial OXPHOS. Nevertheless, rapidly-proliferating cells, such as stem cells or lymphocytes, show a glycolytic metabolism, suggesting that glycolysis can efficiently sustain proliferation even in normal cells [11,12,13,14].

Recently, the hypothesis of mitochondrial involvement in tumour transformation processes has emerged. In particular, the ability of cancer cells to switch from glycolytic to oxidative metabolism seems to be essential to promote their survival. In this scenario, the phenotype of cancer cells is characterized by a remarkable capacity of metabolic reprogramming, which is fundamental for adaptation to different micro-environmental conditions.

Several authors describe the metabolic plasticity of cancer cells as a series of expression waves of metabolic enzymes, which can act simultaneously or sequentially [15].

On the other hand, tumour cells exhibit dependence on glutamine supply, showing overexpression of glutamine transporters [16] and glutaminolysis enzymes [17]. Finally, several studies indicate that essential mitochondrial functions contribute to chemotherapy resistance [18,19,20,21].

Therefore, the study of cellular metabolism adaptation poses remarkable opportunities and challenges to targeted therapy.

Mitochondria are metabolically adaptive organelles, which serve bioenergetic and anabolic functions, hence endowing malignant cells with considerable metabolic plasticity. Beyond bioenergetics, mitochondrial dysfunction can alter the synthesis of several biomolecules to support cancer growth. In cancer cells with mitochondrial impairment, the glutamine-derived intermediates sustain tricarboxylic acid cycle (TCA) and lipogenesis through reductive carboxylation.

Moreover, given that the TCA cycle primarily fuels intermediates for biosynthesis and NADH for the respiratory chain, it is conceivable that alterations in the TCA cycle would take place in response to respiratory chain dysfunction.

The mitochondrial ATP production in different cancer cells ranges from less than 10% in lung epidermoid carcinoma HLF-a cells to some 80% in MCF-7 cells [22,23]. Hence, to target cancer metabolism, it is pivotal to elucidate the dependence of malignancies of cancer cells on mitochondrial functions. To this end, we established a mitochondrial dysfunction in MCF7 breast cancer cells by inhibiting the ubiquinone (CoQ) biosynthesis, aiming to investigate how cancer cell adapts their metabolism to mitochondrial dysfunction. Depletion of CoQ promotes a glycolytic shift of cellular metabolism, driven by hif-1a stabilization, and the onset of oxidative stress. In this paper, we identified a variety of metabolic changes in cancer cells due to CoQ depletion, in particular affecting glycolysis, TCA cycle and glutamine metabolism. Moreover, we found that CoQ depletion decreases the adhesion ability of MCF-7 cells and decrease their capacity to form sphere-shaped aggregates (namely spheroids). Finally, we investigated the effect of glycolysis inhibitors and antiproliferative compounds in tumour cells with or without dysfunctional mitochondria.

## 2. Results

### 2.1. Mitochondrial Dysfunction Induced by CoQ10 Depletion

**Figure 1:**
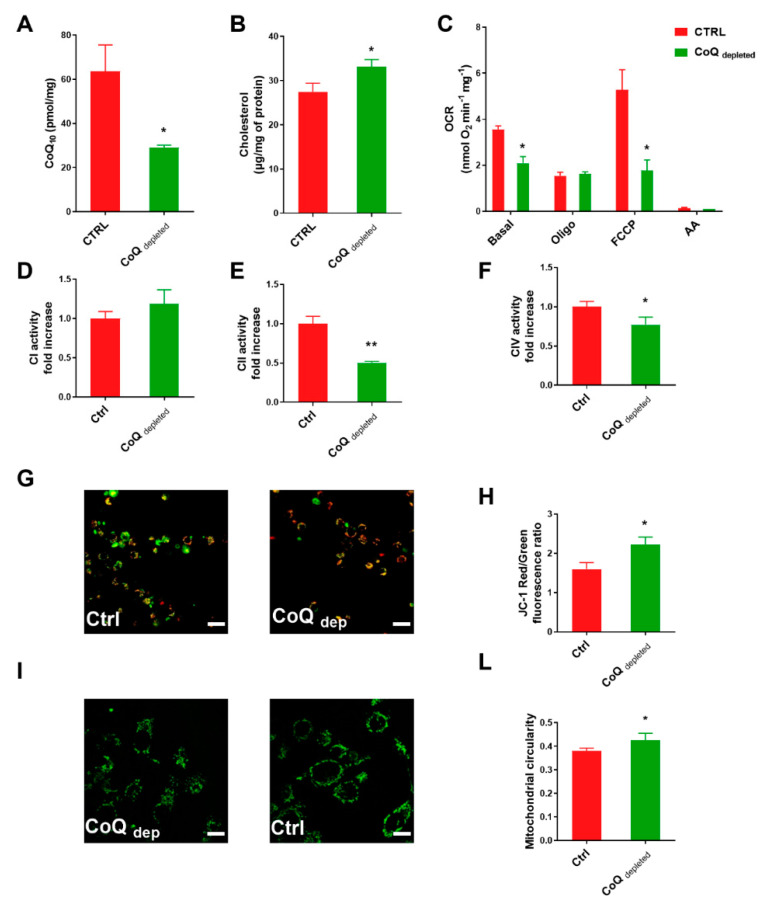
**CoQ depletion induces mitochondrial dysfunction**. MCF-7 cells were cultured in complete DMEM for 96 h in the presence of 4 mM 4-nitrobenzoic acid (CoQ depleted) or vehicle (CTRL). (**A**) Total ubiquinone determination in control and CoQ depleted MCF-7 cells normalized on protein content, (*n* = 3). (**B**) Total cholesterol content in cell lysate from control and CoQ depleted cells normalized on protein content. (**C**) Oxygen consumption rate (OCR) in intact cells measured in DMEM (basal respiration), in the presence of oligomycin A (Oligo) and carbonyl cyanide 4-(trifluoromethoxy) phenylhydrazone (FCCP). To inhibit the mitochondrial respiration, 2 µM Antimycin A was added at the end of each experiment. (*n* = 4). Electron transport chain complex I (**D**), II (**E**), and IV (**F**) enzyme activities were assessed in lysates from control and CoQ depleted cells. Results are displayed as fold increase (*n*  =  3). (**G**) Representative micrographs and their quantification (**H**) of control and CoQ depleted cells stained with JC-1 dye, a cationic dye (green) which exhibits potential-dependent accumulation in mitochondria where it starts forming J aggregates (red). The mitochondrial potential was assessed by measuring the red on green fluorescence intensity ratio. Two randomly chosen fields, from three independent experiments, were analyzed for each condition. Scale bar: 70 µm. (**I**) Representative micrographs of control and CoQ depleted cells stained with Mitotracker green. Mitochondrial circularity value determination (**L**) using ImageJ software. For each condition were analyzed at least two randomly chosen fields from three independent experiments. Scale bar: 47 µm. Statistical analysis was performed using GraphPad Prism software. Error bars indicate the standard error of the mean (SEM). *p* values were obtained using unpaired *t*-test with Welch’s correction. * *p* ≤ 0.05; ** *p* ≤ 0.01.

4-nitrobenzoic acid (4-NB) is a competitive inhibitor of coq2, a prenyltransferase involved in CoQ10 biosynthesis at the mitochondrial level. Figure 1A shows that 4-NB treatment significantly decreased the ubiquinone level in MCF7 cell line by 60%.

In eukaryotic cells, the CoQ10 and cholesterol share the initial part of their biosynthetic pathways. The inhibition of coq2 by 4-NB inhibits CoQ biosynthesis, making available substrates that can indirectly increase cholesterol synthesis [24,25]. We measured the total cholesterol level in MCF7 cells by using HPLC technique, finding that 4-NB treatment increased the cholesterol level in comparison to controls (Figure 1B).

Since CoQ10 is an obligate component of the mitochondrial electron transport chain (ETC), the depletion of CoQ10 pool could directly impair the oxygen consumption rate (OCR). Figure 1C shows that the basal OCR, as well as the spare respiratory capacity (the difference between uncoupled OCR and oligomycin inhibited OCR), were decreased by 4-NB treatment. Moreover, the OCR after oligomycin addition was unchanged, suggesting that 4-NB treatment does not induce proton leaking. The total absence of oxygen consumption in the presence of antimycin A rules out the possibility of extra-mitochondrial O_2_ consumption.

CoQ depleted cells showed low respiration rates, which could be related to an impairment of the electron transfer complexes. To exploit this point, we measured the activity of functionally isolated complex I, complex II and complex IV in MCF7 cells. We found that CoQ depletion did not affect complex I (Figure 1D) and induced only a slight decrease of complex IV activity (Figure 1F); conversely, CoQ depletion dramatically reduced complex II activity (Figure 1E).

Since the mitochondrial transmembrane potential (ΔΨm) is a marker of functionality, we stained the cells with the fluorescent dye JC-1, which exhibits potential-dependent accumulation in mitochondria. Figure 1G,H showed that CoQ depletion induced hyperpolarization in MCF7 cells, indicative of a reduced oxidative phosphorylation activity [26].

Mitochondria are dynamic organelles that can undergo fusion and fission processes resulting in elongated or fragmented morphology. Changes in mitochondrial morphology can influence the cellular bioenergetic status and are implicated in embryonic development, metabolism, apoptosis, and autophagy [27,28,29].

To study the mitochondrial network morphology, we stained the cells with mitotracker green (Figure 1I) and measured the circularity parameter (Figure 1L). Mitochondria exhibiting a perfect circular shape have a circularity value close to 1.0 whereas more elongated mitochondria have a circularity value that is closer to zero [30,31]. In CoQ depleted cells, we found an increased circularity value, compatible with a less elongated mitochondrial shape in comparison with control.

### 2.2. Redox State and Cellular Bioenergetics in CoQ Depleted Cells

Mitochondria are involved in many redox-dependent processes; they produce high amounts of reactive oxygen species (ROS) and present many redox systems, including glutathione (GSH/GSSG) and nicotinamide adenine dinucleotide (NADH/NAD+) reduced/oxidized forms, that are critical for maintaining redox homeostasis [32].

**Figure 2:**
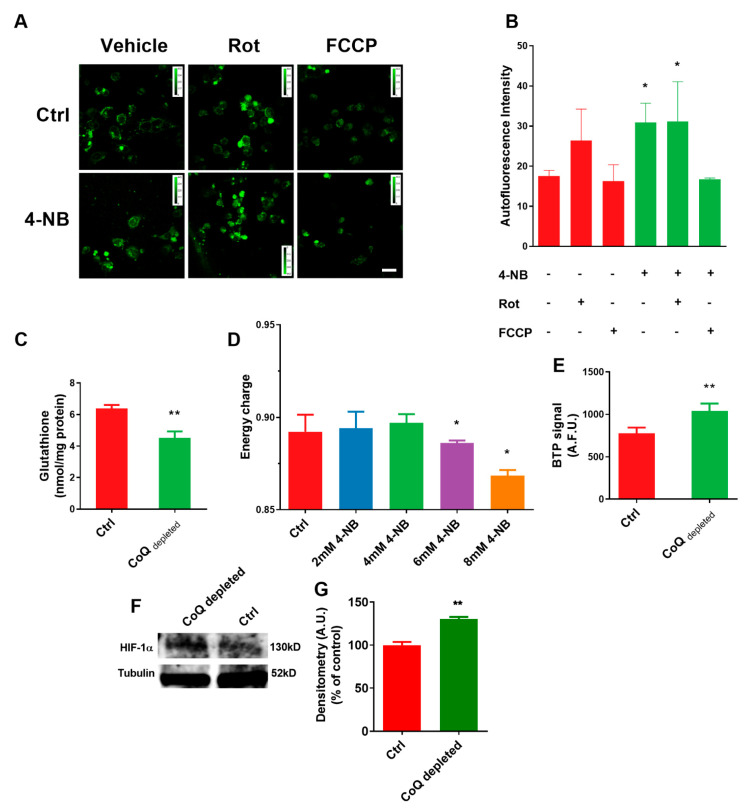
**CoQ depletion alters redox and bioenergetic state in MCF-7 cells.** MCF-7 cells were cultured in complete DMEM for 96 h in the presence of 4 mM 4-nitrobenzoic acid (4-NB) or vehicle (CTRL). (**A**) Representative micrographs and their quantifications (**B**) of NAD(P)H autofluorescence in control and 4-NB treated cells in the presence of 10 µM Rotenone (Rot) or 10 µM carbonyl cyanide 4-(trifluoromethoxy) phenylhydrazone (FCCP). For each condition were analyzed at least two randomly chosen fields from three independent experiments with ImageJ software. Scale bar: 70 µm. (**C**) Determination of reduced glutathione content determination in control and 4-NB treated cells. (**D**) Cellular energy charge quantification in cells cultured for 96 h in the presence of different concentrations of 4-NB (*n* = 3). (**E**) Quantification of fluorescence emission in control and 4-NB treated cells stained with the dye bis (2-(2′-benzothienyl)-pyridinato-N, C30) iridium (acetylacetonate) (BTP). BTP is a live-cell O_2_ sensor which fluorescence emission is quenched by molecular oxygen. (**F**) HIF-1α level in cells lysates. (**G**) Quantification of HIF-1α performed by densitometry analysis, using tubulin as a loading control (*n* = 2). Error bars indicate the standard error of the mean (SEM). Statistical analysis was performed using GraphPad Prism software. For comparison between two groups, *p* values were obtained using unpaired *t*-test with Welch’s correction. For comparison between multiple groups, *p* values were obtained using the analysis of variance (ANOVA) followed by Tukey’s post hoc test. * *p* ≤ 0.05; ** *p* ≤ 0.01.

The NAD+/NADH ratio is a marker of cellular redox state; it regulates catabolic versus anabolic reactions as well as linking the oxidative phosphorylation with the TCA cycle. We measured the NAD(P)H level in cells treated with 4-NB, in the presence/absence of the specific complex I inhibitor rotenone and the uncoupler FCCP. We observed a significant increase of intracellular NADH in CoQ depleted cells in comparison with controls. (Figure 2A,B). Moreover, the NADH level in control cells equalized that of rotenone-treated CoQ depleted cells; this suggests that the build-up of NADH pool in CoQ-depleted cells could be attributable to a block of the respiratory chain, compatible with the increased mitochondrial membrane potential shown in Figure 1L. The addition of FCCP decreased the NADH level in both control and 4-NB-treated cells.

An increased intracellular reducing power together with increased mitochondrial membrane potential could promote electron escape from the respiratory chain generating ROS. Moreover, the depletion of an antioxidant molecule such as the reduced form of CoQ10 could imbalance the ROS homeostasis in CoQ depleted cells. We found that CoQ depletion significantly decreased the GSH level in MCF7 cells (Figure 2C).

The redox imbalance found in CoQ depleted cells strongly suggests an energetic impairment. To investigate this point, we measured the adenylate energetic charge in MCF7 cells using the following equation: ([ATP] + ½ [ADP])/([ATP] + [ADP] + [AMP]) [33].

We found that the energetic charge was unaffected by 4-NB treatment up to 4 mM, while higher concentrations significantly decreased it (Figure 2D).

The cells treated with 4 mM 4-NB maintained the energetic charge despite a reduced OXPHOS; this implies an adaptive metabolic response. We can assume that mitochondrial-defective cells can switch their metabolism towards glycolysis and/or downregulate the energy demand, to cope with impaired oxidative phosphorylation.

Recently, Liparulo et al. [34] reported a metabolic switch toward glycolysis in cells depleted of CoQ, driven by the hypoxia-induced factor HIF-1α. We measured the intracellular oxygen level using an iridium (III) complex dye (BTP). BTP signal is inversely proportional to the intracellular oxygen tension as the dye fluorescence emission is quenched by molecular oxygen. We found that BTP fluorescence intensity was remarkably higher after 4-NB treatment (Figure 2E) indicating that the endogenous oxygen level in CoQ depleted cells was lower than control.

### 2.3. Glucose Uptake and Utilization in CoQ Depleted Cells

**Figure 3:**
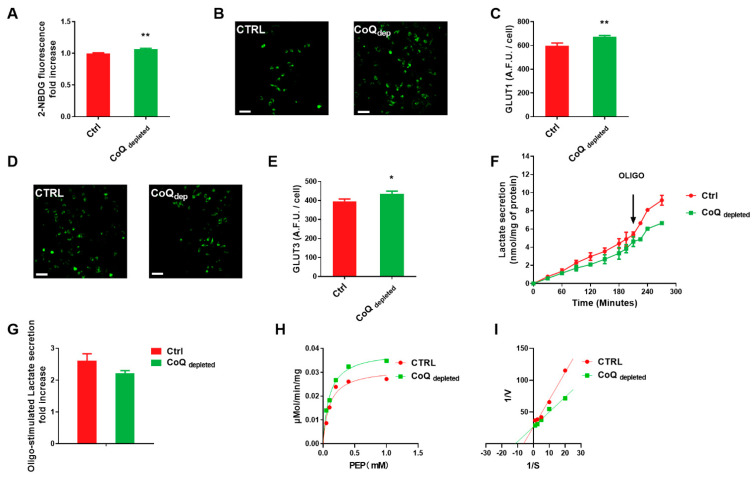
**Glucose uptake and metabolism in CoQ depleted cells.** MCF-7 cells were cultured in complete DMEM for 96 h in the presence of 4 mM 4-nitrobenzoic acid (CoQ depleted) or vehicle (CTRL). (**A**) Glucose uptake determination in MCF-7 cells using the glucose fluorescent analogue 2-deoxy-2-((7-nitro-2,1,3-benzoxadiazol-4-yl)amino)-D-glucose (2-NDBG). (**B**) Representative micrographs and their quantification (**C**) of GLUT1 immunostaining in control and CoQ depleted cells. (**D**) Representative micrographs and their quantification (**E**) of GLUT3 immunostaining in control and CoQ depleted cells. Scale bar: 70 µm. (**F**) Lactate secretion determination in culture medium from control and CoQ depleted cells. 1µM Oligomycin A was added to maximize the lactate production. Lactate was quantified by HPLC and the data were normalized on cellular protein content. (**G**) Fold increase of cellular lactate secretion after oligomycin A addition in control and CoQ depleted cells. (**H**) Michaelis and Menten and (**I**) Lineweaver-Burke plots of pyruvate kinase (PK) activity obtained in the presence of increasing concentrations of phosphoenolpyruvate (PEP) in control and CoQ depleted cells. Statistical analysis was performed using GraphPad Prism software. Error bars indicate the standard error of the mean (SEM). *p* values were obtained using unpaired *t*-test with Welch’s correction. * *p* ≤ 0.05; ** *p* ≤ 0.01.

We assessed the rate of glucose uptake in CoQ depleted cells using the fluorescent glucose analogue (2-NBDG) (Figure 3A). The increased glucose uptake rate accounts for the glycolytic phenotype induced by CoQ depletion. To verify whether an increased glucose uptake mirrored an increased expression of glucose transporters, we evaluated by immunofluorescence the expression of the transporters GLUT1 and GLUT3, which largely contribute to glucose uptake because of their high affinity for the sugar. CoQ depleted cells showed an increased expression of GLUT1 and GLUT3 in comparison to controls (Figure 3C,E).

Increased lactate production is a hallmark of glycolytic metabolism. As shown in Figure 3F, the basal lactate secretion rate was 1.46 ± 0.11 nmol mg^−1^ h^−1^, and 1.03 ± 0.09 nmol mg^−1^ h^−1^ in control and CoQ depleted cells, respectively. The maximal lactate secretion rate, achieved by inhibiting the oxidative phosphorylation with oligomycin A, was 3.78 ± 0.29 nmol mg^−1^ h^−1^ and 2.28 ± 0.21 nmol mg^−1^ h^−1^ in control and CoQ depleted cells, respectively. It is noteworthy that oligomycin treatment induced an increase of the lactate secretion rate compared to the basal value by 260% in control and 220% in CoQ depleted cells (Figure 3G); this suggests that CoQ depletion has compromised the mitochondrial functionality pushing the cells toward a glycolytic metabolism.

Pyruvate kinase (PK) is one of the regulatory enzymes of the glycolytic pathway, which catalyses the conversion from phosphoenolpyruvate (PEP) to pyruvate. Calculating the Km and Vmax parameters of PK from the Michalis-Menten (Figure 3H) and Lineweaver-Burk plots (Figure 3I), we found that the CoQ depletion decreased the Km value of the enzyme, while unaffecting the Vmax. The higher affinity of PK to PEP in 4-NB treated cells suggests an allosteric activation of the enzyme.

### 2.4. Glutaminolysis, Pyruvate Metabolism, and TCA Cycle in CoQ Depleted Cells

OXPHOS-defective cells present increased glutamine anaplerosis. Anaplerotic replenishment of the TCA cycle from glutaminolysis is a two steps conversion. The first step involves the hydrolysis of glutamine, through glutaminase (GLS), and the second implicates the conversion of glutamate to α-KG, through glutamate dehydrogenase (GDH).

**Figure 4:**
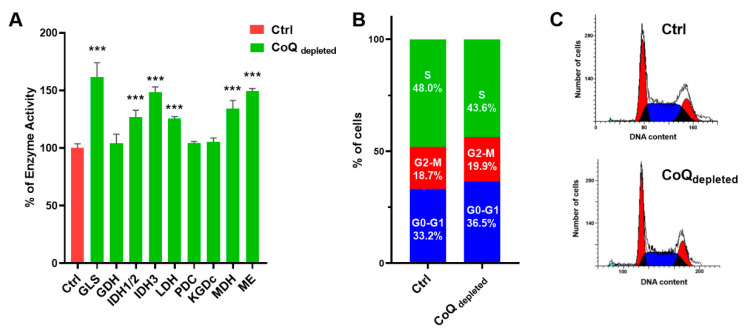
**CoQ depletion induces alteration in enzyme activities and cell cycle.** MCF-7 cells were cultured in complete DMEM for 96 h in the presence of 4 mM 4-nitrobenzoic acid (CoQ depleted) or vehicle (CTRL). (**A**) Enzyme activities (glutaminase, GLS; glutamate dehydrogenase, GDH; isocitrate dehydrogenase1,2 and 3; IDH1/2, IDH3; lactate dehydrogenase LDH; pyruvate dehydrogenase complex, PDC; α-ketoglutarate dehydrogenase, KGDC; malate dehydrogenase, MDH; malic enzyme ME) in control and CoQ depleted cells. Data refers to the percentage increase in comparison with controls. (**C**) Cell cycle analysis and quantification (**B**) by flow cytometry in control and CoQ depleted cells using propidium iodide dye staining. The column graph shows the percentage of cells in each cell cycle. Statistical analysis was performed using GraphPad Prism software. Error bars indicate the standard error of the mean (SEM). *p* values were obtained using the analysis of variance (ANOVA) followed by Tukey’s post hoc test. *** *p* ≤ 0.001.

In Figure 4A, we showed that CoQ depleted cells present a glutaminase (GLS) activity markedly increased, while glutamate dehydrogenase (GDH) activity was unaffected.

Moreover, we found that CoQ depletion induced upregulation of both NAD(P)+-dependent isocitrate dehydrogenase 1 and 2 and NAD+-dependent isocitrate 3 dehydrogenase, as well as malate dehydrogenase (MDH), malic enzyme (ME) and lactate dehydrogenase (LDH). Pyruvate pyruvate dehydrogenase complex (PDC) and α-ketoglutarate dehydrogenase (KGDC) activities were unaffected. The cell cycle analysis showed that CoQ depleted cells arrested in G0/G1 phase, with a concomitant decrease of the S phase (Figure 4B,C).

### 2.5. Effect of Mitochondrial Dysfunction on Cell Proliferation

**Figure 5:**
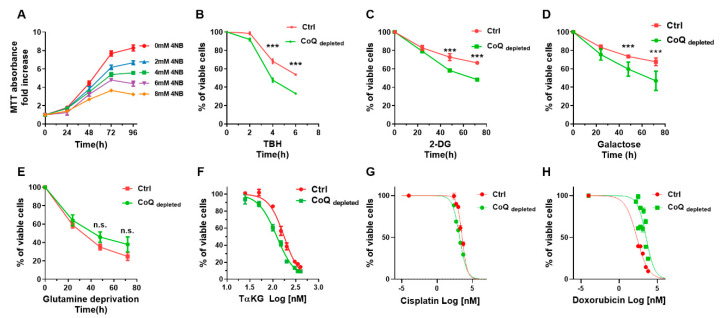
**Effect of mitochondrial dysfunction on cell proliferation.** (**A**) MCF-7 cell proliferation was assessed by MTT assay in the presence of different concentrations of 4-nitrobenzoic acid up to 96 h. (**B**) MCF-7 cells were cultured in complete DMEM for 96 h in the presence of 4 mM 4-nitrobenzoic acid (CoQ depleted) or vehicle (CTRL), then the cells were exposed to 100 µM tert-butyl hydroperoxide (TBH) up to 6 h. (**C**) Control and CoQ depleted cells were cultured in complete medium in the presence of 10 mM hexokinase (HK) inhibitor 2-deoxyglucose (2-DG). (**D**) Control and CoQ depleted cells were cultured in glucose free medium supplemented with dialyzed FBS and 5.5 mM galactose. (**E**) Control and CoQ depleted cells were cultured in a glutamine free complete medium. The cell proliferation in panels B-E was monitored by MTT assay for up to 72 h. (**F**) Control and CoQ depleted cells were cultured in complete medium for 24 h in the presence of different concentrations of trifluoromethyl benzyl-α-ketoglutarate (Tα-KG). Cell proliferation was assessed by MTT assay. (**G**) Control and CoQ depleted cells were cultured in complete medium for 24 h in the presence of different concentrations of doxorubicin and cisplatin (**H**) for 24 h. Cell proliferation was assessed by MTT assay. Statistical analysis was performed using GraphPad Prism software. Error bars indicate the standard error of the mean (SEM). *p* values were obtained using unpaired *t*-test with Welch’s correction. *** *p* ≤ 0.01.

We investigated the effect of ubiquinone depletion on cell growth, finding that 4-NB treatment suppressed MCF7 cell proliferation in a concentration-dependent manner (Figure 5A). Since the CoQ is an antioxidant molecule, we investigated the resistance to oxidative stress of 4-NB treated cells using the radical inducer TBH. Figure 5B shows that 4-NB treatment decreased the viability of the cells exposed to TBH in comparison to controls; this implies that the depletion of CoQ unbalances the oxidative stress homeostasis, making cells unable to counterbalance a further increase in ROS. To assess the reliance on glycolysis of the CoQ depleted cells, we used the competitive glycolysis inhibitor 2-DG (Figure 5C), or we replaced the glucose with galactose in the culture media (Figure 5D), showing that CoQ depletion sensitized the cells both to 2-DG inhibition and galactose treatment, underlining their energy dependence on glycolysis. Conversely, we reported in Figure 5E that CoQ depletion did not sensitize the cells to glutamine deprivation. The BTP fluorescence intensity observed in CoQ depleted cells (Figure 2E) suggests a decreased intracellular oxygen content. Since HIF-1alpha stabilization mediates the survival response to hypoxic stress, we treated the cells with a cell-permeant α-ketoglutarate (TαKG), which increases the degradation of HIF by reactivating the prolyl hydroxylase domain (PHD). From the titration curves obtained treating the cells with TαKG (Figure 5F), we calculated an IC50 value of 176.5 µM and 122.1 µM for control and CoQ depleted cells, respectively; this suggests that cancer cells with mitochondrial dysfunction depend on HIF-1α stabilization. Finally, we tested the sensitivity of CoQ depleted cells to two common anticancer agents: doxorubicin and cisplatin. The results reported in Figure 5G,H show that CoQ depletion does not affect the IC50 for cisplatin (Figure 5G), while increased the IC50 for doxorubicin (Figure 5H).

**Figure 6:**
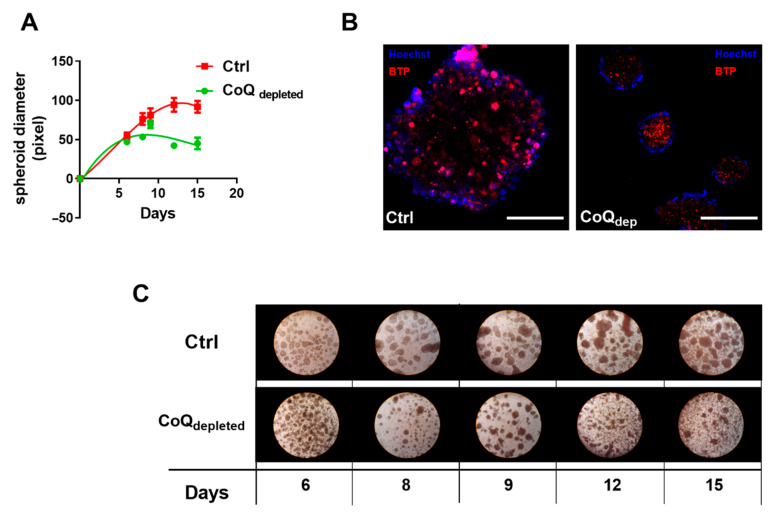
**Effect of CoQ depletion on spheroids formation**. (**A**) Control and CoQ depleted cells were cultured in complete medium and complete medium supplemented with 4 mM 4-NB respectively in high Bioinert Ibidi dishes to allow spheroids formation. The diameter of the spheroid was monitored for 15 days and measured using ImageJ software. (**B**) Representative micrographs of control and CoQ-depleted spheroids stained with Hoechst and the oxygen sensitive probe BTP. Bar size = 100 µm. (**C**) Representative phase contrast light micrographs of control and CoQ-depleted spheroids at different time points.

Since the metabolism of cells cultured in 3D seems to have different active and inactive pathways in comparison with 2D cultures, we investigated the ability to form spheroids in CoQ depleted cells. To this end, we growth control and CoQ depleted cells for 15 days in dishes with non-adherent surface, finding that CoQ depletion significantly hindered the formation of spheroids (Figure 6A,C). Moreover, we stained the spheroids with the oxygen sensitive probe BTP, finding that CoQ-depleted spheroids were in a more hypoxic state in in comparison to controls (Figure 6B).

## 3. Discussion

Cancer cells are endowed with high metabolic plasticity to proliferate in hostile environments. The glycolytic phenotype of cancer cells described by Otto Warburg (namely Warburg effect) represents only a stage of the metabolic reprogramming necessary to tumour cells for growth. Recent studies highlighted the role of mitochondria in tumorigenesis, suggesting that concomitant inhibition of glycolysis and oxidative phosphorylation (OXPHOS) could result in more effective suppression of the cancer cells [35,36]. The “waves” theory of gene expression for the metabolic reprogramming [15] claims that, during malignant transformation, the cells can change their metabolism to cope with nutrient shortage; in the glycolytic shift, which characterizes the first “reprogramming wave”, the pyruvate is partially diverted from OXPHOS to generate biosynthetic precursor required for proliferation. On the other hand, the reduction of OXPHOS activity lowers ROS production, protecting cancer cells from oxidative damage and apoptotic cell death [37].

We studied the effect of mitochondrial impairment in a human breast cancer cell line (MCF-7) in which OXPHOS gives a high contribution to ATP production [23]. The mitochondrial dysfunction was induced by inhibiting the biosynthesis of CoQ using 4-nitrobenzoate (4-NB), an inhibitor of the biosynthetic enzyme coq2 [38]. CoQ is a lipid-soluble molecule present in all cell membranes where it exerts several functions, the most important of which are the transfer of electrons in the mitochondrial respiratory chain and the antioxidant activity. Therefore, the CoQ depletion has two effects: it decreases the ATP level, by impairing electron transfer flow, and increases the oxidative stress. In a recent paper [34], using a different cell model, we described that CoQ depletion induces alterations on respiratory capacity and redox state, which are associated to an increased level of cholesterol and decreased level of intracellular oxygen content. Altogether, these factors contribute to transcription factor HIF-1α stabilization, which drives the metabolic rearrangement necessary to allow cells survivals in these conditions.

In this paper, we analyzed the bioenergetic profile of CoQ depleted MCF-7 cells, confirming the presence of reduced oxygen consumption rate, especially in the uncoupled state, increased membrane potential (mtΔΨ), increased level of ROS, and altered mitochondrial morphology.

A deeper analysis of the respiratory chain activity showed that Complex I activity was unchanged, while complex II and complex IV activities were significantly reduced. Since CoQ acts as substrate only for Complex I and II, the decreased activity of Complex IV could be attributed to enzyme damage induced by the increased ROS and nitric oxide production [34]. Interestingly, a lowered succinate dehydrogenase activity in mitochondria with high transmembrane potential is a well-known phenomenon due to oxaloacetate inhibition, as recently elucidated by Fink B.D. et al. [39].

In CoQ depleted cells, the intracellular oxygen level was lower than controls, despite their reduced oxygen consumption rate. Moreover, the total cholesterol content and the ROS production were increased, while the antioxidant defences, evaluated as reduced glutathione content, were decreased; these parameters are indicative of a metabolic change in cellular metabolism and can stabilize HIF 1α, which in turn pushes cell metabolism toward glycolysis [34]. Since high levels of α-ketoglutarate can destabilize HIF-1α, we supplemented the cells with a membrane-permeable derivative of this compound (TαKG), showing that CoQ depleted cells viability was significantly affected. The latter finding strongly suggested that CoQ-depleted cells were dependent for their survival on the activation of the genes involved in the hypoxic response, elicited by HIF 1α.

Interestingly, HIF-1a is directly affected by the activity of the respiratory complex II, since the succinate inhibits the prolyl-hydroxylase domain enzymes (PHDs) activity, leading to HIF-1α stabilization under normoxic conditions [40,41]. Remarkably, the MCF-7 cells treated with 4-NB showed a 50% decrease in complex II activity, suggesting that its impairment could be significant in the metabolic reprogramming in cells with mitochondrial dysfunction.

It is reported that cancer cells with attenuated cellular respiration depend on glycolysis for energy production [42]. To test the reliance of CoQ-depleted cells on glycolysis, we replaced the glucose in the culture medium with galactose [43,44], finding that galactose treatment significantly decreased their growth rate in comparison with controls. However, in the presence of glucose, the glycolytic metabolic switch can efficiently compensate for the lack of energy due to OXPHOS impairment. In fact, the total energetic charge of CoQ-depleted cells in the presence of glucose was similar to controls.

CoQ depleted cells showed a higher glucose uptake associated with a higher expression of glucose transporters Glut 1 and Glut 3 in comparison with controls. Unexpectedly, despite the glycolytic metabolism, CoQ-depleted cells showed a lower basal lactate secretion rate. To elucidate this point, we measured the lactate secretion induced by ATP synthase inhibition, using oligomycin A. The cells with functional mitochondria respond to oligomycin A inhibition with a strong increase in lactate secretion, while in cells with defective mitochondria, the stimulation of lactate secretion is low [45]. In MCF-7 cells treated with 4-NB, the release of lactate induced by oligomycin A was lower in comparison to control cells, confirming their glycolytic metabolism.

The metabolic rearrangement induced by CoQ depletion involves the activation/inactivation of enzymes of several metabolic pathways. We found that pyruvate kinase showed decreased Km for phosphoenolpyruvate (PEP), suggesting an allosteric activation. On the other hand, the expression of pyruvate dehydrogenase was unaffected, while the lactate dehydrogenase was increased; these modifications are consistent with increased glycolytic flux, without a concomitant enhancement of the oxidative degradation of pyruvate. In CoQ depleted cells, pyruvate could be partially redirected toward biosynthetic pathways, as suggested by the low lactate secretion rate and the increased activity of LDH; nevertheless, this point requires further experiment to be elucidated.

Analyzing the activities of Krebs’s cycle enzymes, we found increased activities of glutaminase, isocitrate dehydrogenase isoforms, and malate dehydrogenase, as well as the malic enzyme. Since CoQ depletion strongly reduces the OXPHOS activity, an increased activity of these enzymes could be explained by considering a reductive metabolism of glutamine, as recently reported by Chen et al. [46]. The stimulation of the reductive carboxylation of α-ketoglutarate derived from glutamine allows cells to provide intermediates for the biosynthetic pathways. Notably, normal and CoQ depleted cells are equally sensitive to glutamine deprivation; the lack of difference could be due to the lower proliferation rate of 4-NB treated cells.

According to the low proliferation rate measured in CoQ-depleted cells, we analysed the cell cycle of CoQ-depleted cells, finding a cell accumulation in G0/G1-phase and a decreased cell number in S-phase.

In a recent paper Gomes A. et al. [47] demonstrated that the cell proliferation rate in a 3D tumour spheroid model was strongly inhibited at low oxygen pressure. In particular, they showed that in spheroids cultured at 5% oxygen concentration, the external layer proliferation was clearly limited at the day 6, while spheroids cultured at 21% oxygen concentration were still able to grow.

Notably, the oxygen reduction affects only 3D spheroids growth and has almost no effect on the 2D cell culture. Moreover, these effects are induced by an oxygen concentration of 5%, which is not considered a hypoxic condition. Since we found that CoQ depleted cells were characterized by a lower intracellular oxygen concentration, we analyzed the ability of CoQ depleted and control cells to form 3D structure. The results reported in Figure 6 demonstrated that the dimensions of spheroids were similar until the day 8–10. After this time, 3D spheroids derived from control cells increased their size, while spheroids derived from CoQ-depleted cells stopped their grown. Moreover, we found that oxygen distribution inside spheroids was different: spheroids derived from control cells presented a hypoxic centre, while in spheroids derived from CoQ-depleted cells the hypoxic region was extended up to the outer layer of the structure. These results confirm the role of intracellular oxygen concentration as major rate limiting factor for cell proliferation rate.

It is known that hypoxia contributes to tumour resistance to radio- and chemo- therapy: here we reported that CoQ-depleted MCF-7 cells were more resistant to doxorubicin. A recent study has pointed out that increased mitochondrial cholesterol can contribute to chemotherapy resistance [48]. Thus, it is likely that elevated cholesterol reduced susceptibility to doxorubicin in cancer cells treated with 4NB. It is also plausible that deregulation of intrinsic apoptosis pathway may account for this resistance [49].

In conclusion, CoQ-depleted cells showed a decreased proliferation rate, an increased sensitivity to oxidative stress injury and energy reliance on glycolysis. Moreover, CoQ-depleted cells showed a low intracellular oxygen concentration, which hinders the formation of 3D spheroids. These results shed light on the mechanisms of tumor metabolic reprogramming provided an insightful understanding of cancer metabolism targeting.

## 4. Materials and Methods

### 4.1. Cell Culture and CoQ Depletion

MCF7 breast cancer cells (ATCC, Manassas, VA, USA) were cultured in Dulbecco’s Modified Eagle Medium (DMEM, high glucose) supplemented with 10% fetal bovine serum (FBS), and 2 mM glutamine. Cells were grown at 37 °C and 5% CO_2_ in a humidified cell culture incubator. If not diversely mentioned, CoQ depletion was achieved by growing cells for four days in complete DMEM supplemented with 4 mM 4-nitrobenzoic acid (4-NB).

### 4.2. Coenzyme Q Determination

Coenzyme Q extraction was performed as described by Takada et al. [50]. CoQ was quantified by HPLC according to Bergamini et al. [51].

### 4.3. Cholesterol Assay

The total cholesterol content of cells was quantified by HPLC according to Contreras et al. [52] with minor modifications [34]. External cholesterol standards were used for calibration. All the samples were analysed in triplicate.

### 4.4. Polarographic Assay

Cells were detached by trypsinization, washed with phosphate-buffered saline, and assayed for oxygen consumption at 37 °C in DMEM using an oxygraph chamber (Instech Mod. 203, Plymouth Meeting, PA, USA). The oxygen consumption rates were measured in DMEM (endogenous respiration) and in the presence of 1 µM oligomycin A and 0.1–1 µM carbonyl cyanide 4-(trifluoromethoxy) phenylhydrazone (FCCP). To inhibit the mitochondrial respiration and to rule out extramitochondrial oxygen consumption, we added 5 µM antimycin A at the end of the experiments. Data were normalized on protein content [53]. All experiments were performed in triplicate.

### 4.5. Assessment of Respiratory Complexes

The mitochondrial complex I and complex II activities were measured following Spinazzi et al. [54] with minor modifications. Experiments were performed at least in triplicate. Complex IV (cytochrome c oxidase) activity was determined following Barrientos et al. [55]. Briefly, cells were harvested and resuspended in respiratory buffer (RB): 0.3 M mannitol, 10 mM KCl, 5 mM MgCl_2_, and 10 mM K_2_PO_4_, pH 7.4. Then, the cell suspension was injected into the polarographic chamber (1.5 × 106 cell/mL) and the reaction was started by adding ascorbate (10 mM) plus N,N,N′,N′-tetramethyl-p-phenylenediamine (TMPD, 0.2 mM). The oxygen consumption was recorded for 2–5 min and finally, KCN (700 µM) was added to block complex IV activity. The final respiratory rate is obtained by subtracting the KCN-insensitive respiration. Data were normalized to the cellular protein content determined by the Lowry method [53].

### 4.6. Mitochondrial Morphology Analysis

Cells were seeded and cultured in 15-well μ-slides (Ibidi, GmbH, Planegg/Martinsried, Germany).) according to manufacturer instructions. For mitochondrial staining, cells were carefully washed with PBS and loaded with 100 nM Mitotracker Green, MTG (λexc 490 nm, λem 516 nm; Thermo Fisher Scientific, Waltham, MA, USA) according to Chazotte B [56]. Images were acquired on a Nikon C1si confocal microscope (Nikon, Tokyo, Japan). Analysis of mitochondrial morphology was performed using ImageJ standard tools. The circularity of the objects was employed as an index of mitochondrial fragmentation. A circularity value of 1.0 indicates a perfect circle. As the value approaches 0.0, it indicates an increasingly elongated polygon.

### 4.7. Mitochondrial Membrane Potential Determination

To measure mitochondrial membrane potential, cells were stained with the fluorescent dye JC-1 ((5, 5′, 6, 6′-tetrachloro-1, 1′, 3, 3′-tetraethylbenzimidazol carbocyanine iodide) (Thermo Fisher Scientific, Waltham, MA, USA) and analyzed by confocal microscopy following Sivandzade et al. [57]. Briefly, MCF-7 cells were grown on coverslips and stained for 30 min with 5 µM JC-1 dissolved in the culture medium. After this time, cells were carefully washed with phosphate saline buffer and visualized by confocal microscopy. At least five randomly chosen fields for each condition were analyzed. Fluorescence intensity was quantified by Image J software (NIH).

### 4.8. Measurement of Intracellular NAD(P)H

NADH autofluorescence measurements were performed following Frezza et al. [7] with minor modifications. NAD(P)H autofluorescence were acquired in the presence of 10 µM Rotenone to completely block mitochondrial complex I NADH consumption and in the presence of 10 µM FCCP to maximize NADH oxidation by the respiratory chain. For each condition, at least five randomly selected fields were analyzed. After background subtraction, fluorescence intensity quantification was performed using ImageJ software.

### 4.9. Adenine Nucleotides Measurement and Energy Charge Determination

Cellular adenine nucleotides were extracted and detected by HPLC following Jones [8] using a Kinetex C18 Column (250 Å~ 4.6 mm, 100 A°, 5 μm; Phenomenex, Torrance, CA, USA). ATP, ADP and AMP peaks were identified by comparison and co-elution with the standards. The quantification was obtained by peak area measurement using standard curves. The adenylate energy charge was calculated using the equation: ([ATP] + ½ [ADP])/([ATP] + [ADP] + [AMP]) [9].

### 4.10. Glutathione Determination

Intracellular glutathione (GSH) level was measured by HPLC after derivatization with N-ethylmaleimide (NEM) following Giustarini et al. [58].

### 4.11. Measurement of Intracellular Oxygen

Intracellular oxygen tension was measured in intact cells following Liparulo et al. [34], using 5 µM of BTP (bis(2-(2′-benzothienyl)-pyridinato-N,C3′)iridium(acetylacetonate) (Sigma-Aldrich, St. Louis, Missouri, MO, USA), a fluorescent dye which light emission is quenched by molecular oxygen [59]. For three-dimensional cell culture 8 µM of Hoechst (Sigma-Aldrich, St. Louis, Missouri, MO, USA) was used to stain nuclei. 

### 4.12. Immunofluorescence Staining of GLUT1 and GLUT3 and Glucose Uptake Assay

GLUT1 and GLUT3 transporters expression was measured by immunofluorescence. MCF7 cells were grown on glass coverslips and exposed to 4-NB for 72 h. Then, cells were fixed, permeabilized and incubated with goat polyclonal primary antibodies against Glut1, or Glut3, (Santa Cruz Biotechnology, Dallas, TX, USA), overnight at 4 °C, followed by appropriate anti-goat-FITC secondary antibodies. Specimens have been embedded in Mowiol (Hoechst, Frankfurt, Germany) and multiple images acquired by using sequential laser excitations at 488 nm to reduce spectral bleed-through artifacts. The images have been collected by using a Nikon C1s confocal laser-scanning microscope, equipped with a Nikon PlanApo 60X, 1.4-NA oil immersion lens. To determine cellular glucose uptake, the fluorescent glucose analogue 2-[N-(7-nitrobenz-2-oxa-1,3-diazol-4-yl)amino]-2-deoxy-d-glucose (2-NBDG; Thermo Fisher Scientific, Waltham, MA, USA) was used following Liparulo et al. [34].

### 4.13. Western Blot Analysis

Western blot analysis of HIF-1α was performed following Liparulo et al. [34].

### 4.14. Lactate Determination

Basal lactate secretion rate in live cells was analyzed by measuring the lactate content in the culture medium by HPLC analysis. 1 μM oligomycin A was used to achieve the maximal lactate secretion rate by blocking the mitochondrial ATPase. For lactate quantification, the culture medium was diluted 1: 10 in 50 mM KH_2_PO_4_ pH 2.4 and centrifuged at 14,000× *g* for 5 min at 4 °C. The lactate content in the supernatant was quantified on an HPLC system (Agilent 1100 Series System) equipped with a phenylic column (Agilent ZORBAX SB-Phenyl, 5 μm, 250 Å, 4.6 mm). The mobile phase consisted of 50 mM KH_2_PO_4,_ pH 2.4; the flow rate was 0.8 mL min−1. Absorbance was monitored at λ210 nm by a photodiode array detector. The quantification of lactate was obtained by peak area measurement compared with standard curves. All injections were performed in triplicate.

### 4.15. Pyruvate Kinase (PK) Activity

Pyruvate kinase activity was measured by a continuous assay coupled to lactate dehydrogenase (LDH) [60]. NADH absorbance was followed spectrophotometrically at 340 nm (ε340 nm = 6220 M−1 cm−1) using a spectrophotometer (Jasco V-550) equipped with thermostatic control and stirring device. Kinetic assays for activity determinations contained cell lysate (1–2 μg), Tris pH 7.5 (50 mM), KCl (100 mM), MgCl2 (5 mM), ADP (0.6 mM), NADH (180 μM) and LDH (8 units). Different concentrations of phosphoenolpyruvate (PEP) ranging from 0 to 1.5 mM were added to initiate the reactions. Michaelis-Menten plot and Lineweaver-Burk plot were used to analyze the Vmax and Km of PK.

### 4.16. NAD+-Dependent and NADP+-Dependent Enzyme Activity

NAD+ and NADP+ dependent enzyme activity were measured in cell lysates following resorufin fluorescence emission (λexc 550 nm; λem 590 nm). The reaction was performed in a 96-well microplate and measured using a with a multi-plate reader (EnSpire; PerkinElmer). Briefly, 50 µg of cell lysate was mixed with 1 mM MgCl_2_, 0.1 mM CaCl_2_, 3 mM NAD+ or NADP+, 10 µM rotenone and 200 µM resazurin in 50 mM MOPS buffer (pH 7.4). Then, 10 mM of specific substrates of lactate dehydrogenase (LDH), pyruvate dehydrogenase (PDH), NADP+-dependent isocitrate dehydrogenase (IDH), NAD+-dependent isocitrate dehydrogenase (IDH), α-ketoglutarate dehydrogenase (KGDH), malate dehydrogenase (MDH), malic enzyme (ME), glutamate dehydrogenase (GDH) and glutaminase (GLS) were added to start the reactions. The linear part of the product accumulation curves was used for the reaction rate determination. In each experiment, fluorescence from four wells was averaged and NAD(P)H:resazurin oxidoreductase rates of the CoQ depleted cells were expressed as a percentage of the rates exhibited by the control cells.

### 4.17. Cell Cycle Analysis

Control and CoQ depleted cells were seeded in T25 flasks at a density of 20,000 cells/cm^2^ in complete medium or complete medium supplemented with 4 mM 4-NB respectively for four days. After this time, cells were detached by trypsinization, washed in PBS, and collected by centrifugation. The pellet was resuspended in 10 μg/mL RNase, 0.1% sodium citrate, 0.01% Nonidet P-40, and 50 μg/mL propidium iodide (PI) for 30 min in the dark. PI fluorescence emission was analyzed on a Beckman Coulter Epics XL-MCL flow cytometer. The cell cycle analysis was performed using the M cycle (Verity) and MODFIT 5.0 software [61].

### 4.18. Spheroids Formation Assay

To test the effect of CoQ depletion on spheroids formation, MCF-7 cells were seeded at 0.1 × 106 density in µ-Dish 35 mm, high Bioinert (Ibidi, Germany), in complete medium and complete medium supplemented with 4 mM 4-NB respectively. The size of the spheroids was monitored up to 15 days by measuring their diameter using ImageJ software.

Spheroids formation assay. For spheroids formation the suspension method described by Froehlich et al. [62] was followed with minor modifications. To test the effect of CoQ depletion on spheroids formation, MCF-7 cells were seeded at 0.1 × 106 density in µ-Dish 35 mm, high Bioinert (Ibidi, Germany), in complete medium with 25% of Methocel (Sigma-Aldrich, St. Louis, Missouri, MO, USA). For CoQ-depleted cells the medium was supplemented with 4 mM 4-NB. The size of the spheroids was monitored up to 15 days by measuring their diameter using ImageJ software.

### 4.19. Cell Viability Assays

For cell viability tests, control and CoQ depleted cells were seeded at 20,000 cells per well in a 96-well plate in complete DMEM and complete DMEM plus 4 mM 4-NB respectively. Cells were incubated at 37 °C and 5% CO_2_ for 24 h to allow adhesion. To test the effect of tert-butyl hydroperoxide (TBH) exposure, the complete culture medium was replaced with culture medium plus 100 µM TBH. Cell viability was measured after 2 h, 4 h, and 8 h respectively by MTT assay. Accordingly, to test the effect of glycolysis inhibition 10 mM hexokinase 2-deoxyglucose (2-DG), a hexokinase (HK) inhibitor, was used. Cell viability was measured at 24 h, 48 h, and 72 h after addition of 2-DG by MTT assay.

To test the energetic reliance of cells to oxidative phosphorylation, the complete culture medium was replaced with glucose-free DMEM supplemented with 10% dialyzed FBS and 5.5 mM galactose. To test the reliance on glutamine supplementation, the complete culture medium was replaced by glutamine-free DMEM and 10% dialyzed FBS. Cell viability was assessed up to 72 h of treatment by MTT assay.

To test the effect α-ketoglutarate on control and CoQ depleted cells viability, we treated the cells with different concentrations of a cell-permeant alpha-ketoglutarate (trifluoromethyl-benzyl alpha-ketoglutarate; Tα-KG) [63]. Cell viability was assessed after 24 h of treatment by MTT assay.

To test the effect of doxorubicin and cisplatin on control and CoQ depleted cell viability, we treated the cells with different concentrations of compounds and the cell viability was assessed after 24 h by MTT assay.

### 4.20. Statistical Analysis

Statistical analysis was performed using GraphPad software. The number n refers to the number of biological replicates.

## Data Availability

Data are contained within the article.

## Abbreviations

GLUT1	Glucose Transporter Type 1
GLUT3	Glucose Transporter Type 1
CoQ	Coenzyme Q
OXPHOS	Oxidative phosphorylation
NADH	Nicotinamide adenine dinucleotide
ETC	Electron transport chain
OCR	Oxygen consumption rate
4-NB	4-nitrobenzoate
FCCP	Carbonyl cyanide 4-(trifluoromethoxy) phenylhydrazone
ATP	Adenosine triphosphate
ADP	Adenosine diphosphate
AMP	Adenosine monophosphate
PK	Pyruvate kinase
PEP	Phosphoenolpyruvate
PDC	Pyruvate dehydrogenase complex
LDH	Lactate dehydrogenase
ME	Malic enzyme
MDH	malate dehydrogenase
